# SAR Target Configuration Recognition via Product Sparse Representation

**DOI:** 10.3390/s18103535

**Published:** 2018-10-19

**Authors:** Ming Liu, Shichao Chen, Fugang Lu, Mengdao Xing

**Affiliations:** 1 Key Laboratory of Modern Teaching Technology, Ministry of Education, Xi’an 710062, China; mliu@snnu.edu.cn; 2 School of Computer Science, Shaanxi Normal University, Xi’an 710119, China; 3 No. 203 Research Institute of China Ordnance Industries, Xi’an 710065, China; lufugang203@163.com; 4 National Laboratory of Radar Signal Processing, Xidian University, Xi’an 710071, China; xmd@xidian.edu.cn

**Keywords:** synthetic aperture radar (SAR), sparse representation (SR), product model, target configuration recognition

## Abstract

Sparse representation (SR) has been verified to be an effective tool for pattern recognition. Considering the multiplicative speckle noise in synthetic aperture radar (SAR) images, a product sparse representation (PSR) algorithm is proposed to achieve SAR target configuration recognition. To extract the essential characteristics of SAR images, the product model is utilized to describe SAR images. The advantages of sparse representation and the product model are combined to realize a more accurate sparse representation of the SAR image. Moreover, in order to weaken the influences of the speckle noise on recognition, the speckle noise of SAR images is modeled by the Gamma distribution, and the sparse vector of the SAR image is obtained from q statistical standpoint. Experiments are conducted on the moving and stationary target acquisition and recognition (MSTAR) database. The experimental results validate the effectiveness and robustness of the proposed algorithm, which can achieve higher recognition rates than some of the state-of-the-art algorithms under different circumstances.

## 1. Introduction

As one of the most popular application areas of synthetic aperture radar (SAR), SAR target recognition has been deeply exploited due to its great importance in both civil and military areas.

SAR target recognition algorithms have been extensively studied in recent years [1,2,3]. The algorithms can be generally categorized into template-based ones and model-based ones [4]. Template-based algorithms [5,6] are relatively easy to comprehend, but they require a huge storage space. Besides, the computation burden is heavy, especially when the size of the training sample is large.

Owing to the multiple advantages like low storage and computation requirement, robustness to noisy conditions, various model-based algorithms have been proposed for SAR target recognition. Some model-based algorithms construct physical models of the targets (e.g., the scattering center model [7,8], the attributed scattering center (ASC) model [9,10], and the high range resolution profile (HRRP) [11,12]) which can achieve satisfying recognition results, but they heavily depend on the accuracy of the construction of the target models. Besides, some of these algorithms need to estimate the aspect angle of the target first, which is hard to realize [13]. Some other model-based algorithms utilizing effective features of the target to realize target recognition, such as the geometrical properties (e.g., the shadow of the target [14], the length of the target [15], the contour of the target [16]) and the mathematical features (e.g., the non-negative matrix factorization (NMF) [17], the complex wavelet transform [18], the Krawtchouk moments [19], the Zernike moments [4], the scale invariant feature transform (SIFT) feature [20]) have also been proposed. However, these algorithms heavily depend on the effectiveness of feature extraction.

Deep learning algorithms [21,22,23,24] have been popular nowadays, and they have achieved exciting recognition results [22,23,24]. However, not only is the parameter estimation of these algorithms complex, but the computation complexity is heavy as well. Moreover, these algorithms usually suffer from local minimum and overfitting problems [25,26].

Due to the natural discriminative power, sparse representation (SR) has been widely used in pattern recognition fields [27,28,29]. Lots of SR-based algorithms have been proposed for SAR target recognition ever since its first successful application to recognize targets in SAR images by J. Thiagarajan, et al. [30]. Making use of the multi-view SAR images, a joint SR (JSR) algorithm has been proposed by Zhang et al. [31], based on which some modified algorithms have subsequently emerged [32,33]. A series of SR-based SAR target recognition algorithms have been presented utilizing the monogenic feature of SAR images by Dong et al. [34,35,36]. Pan et al. proposed a reweighted sparse representation algorithm to achieve airplane recognition in SAR images [37]. Recently, algorithms combining other powerful techniques with SR have attracted increasing interest, such as the support vector machine [38], the statistical model [39], the label information [40], and so on.

For SR-based recognition algorithms, a key point is to obtain the sparse vector of the SAR image. In many presented SAR target recognition algorithms, the sparse vector is usually calculated through convex optimization [34,36,41,42] or in a greedy way [30,31,38,39] just as the algorithms suitable for the optical images based recognition [28,29]. However, different from the noise in optical images, the speckle noise in SAR images is neither Gaussian nor additive [43]. Due to the coherent imaging mechanism of SAR, the speckle noise in SAR images is multiplicative [44,45,46,47]. As a result, the product model has been widely used to describe SAR images. Quite a few product model distributions have been proposed in different application fields to improve the performance of the algorithms [46]. Motivated by these algorithms, in this paper, we describe SAR images by the product model to better realize SAR target configuration recognition. For the distribution of the speckle component, the Gamma distribution has been widely studied, and it can describe the essential characteristics of SAR images very well [44,45,46,47,48]. So as to describe SAR images in a more accurate way and obtain the essential characteristics of SAR images, in this paper, we describe SAR images by the product model [44,45,46] and try to solve the sparse vectors from a statistical standpoint. The speckle noise of SAR images is modeled by the Gamma distribution [44,45,46,47,48]. Fusing the advantages into SR, the discriminative power can be further enhanced. A more accurate description of the SAR image will lead to better recognition results.

Different from the traditional target recognition algorithms, which focus on the classification of different types, the proposed algorithm tries to challenge a more severe case—target configuration recognition. The configuration of a target indicates the tiny differences among the same target type (for instance, the same target with/without machine guns or fuel barrels). Configuration recognition is of crucial importance in fields which need detailed information capture of the targets, such as battlefield interpretation, reconnaissance, and precise attacking [39,40].

This paper is organized as follows. In Section 2, the proposed product sparse representation (PSR) algorithm is discussed in detail. In Section 3, the effectiveness and robustness of the proposed PSR algorithm are tested on several target recognition experiments using the moving and stationary target acquisition and recognition (MSTAR) database. In Section 4, some conclusions are drawn finally.

## 2. Product Sparse Representation (PSR) for SAR Target Configuration Recognition

As is known, SAR images suffer from speckles due to the imaging mechanism of SAR. Additionally, SAR images can be properly described by the product model. That is to say, for a given sample y (SAR image after dimensionality reduction), we have [44,45,46]

(1) y=R∘ε

where R is the radar cross section (RCS) coefficients of the clutter, ε is the speckle components of the SAR image, and the symbol “∘” represents the element multiplication. SAR images can be better described by using the product model in theory [44,45,46].

SR has been proved to be powerful in pattern recognition fields including SAR target recognition [27,28,29,30,31,32,33,34,35,36,37,38,39,40]. Considering the advantages of SR and the product model, we try to combine them together to get a more accurate sparse description of the SAR image in this paper. The main idea of the proposed method is to describe the SAR images as accurate as possible from a statistical standpoint, and then to enhance the robustness to the speckle in SAR images.

Firstly, we divide the dictionary D consists of all the training samples containing various targets into separated ones. $D_{i}\left(i=1,2,\ldots ,S\right)$ represents the ith dictionary whose samples all belong to configuration i, S is the total number of target classes, and the total number of the samples is $N_{i}$. Defining $R_{i}=D_{i}\alpha_{i}$, $\alpha_{i}$ is the corresponding sparse vector of the testing sample under dictionary $D_{i}$, Equation (1) can be rewritten as

(2) $$y=D_{i}\alpha_{i}\circ \varepsilon_{i}$$

where $\varepsilon_{i}$ represents the speckle of the SAR image corresponding to $D_{i}$.

Similar to all the SR-based recognition algorithms, the key point of the proposed algorithm is to solve the sparse vector of the testing sample accurately. Different from the traditional algorithms [30,31,34,36,38,39] in which the sparse vector is obtained by using the convex optimization algorithms or in a greedy way, we try to get the representation from the statistical view to better describe the SAR image and to enhance the robustness of the proposed algorithm to speckle. To realize accurate SAR target configuration recognition, we expect the obtained $\alpha_{i}$ can give a precise description of the testing sample. From the statistical view, we would like to get the most probable sparse vector of the testing sample. The objective function can be written as

(3) $$J_{i}=\underset{\alpha_{i}}{\arg\max}\;p\left(\alpha_{i}|y\right)$$

Using the Bayesian rule, we have

(4) $$J_{i}=\underset{\alpha_{i}}{\arg\max}\;p\left(y|\alpha_{i}\right)p\left(\alpha_{i}\right)$$

What follows is to model the likelihood function $p\left(y|\alpha_{i}\right)$ and the prior function $p\left(\alpha_{i}\right)$, respectively.

From Equation (2), we can tell that $p\left(y|\alpha_{i}\right)$ shares the same distribution with the speckle. There are several models that can be employed to describe the speckle component, among which the Gamma distribution [44,45,46,47,48] has been widely used. In view of statistics, the speckle component n of the SAR image can be given by [44,45,46,47]

(5) $$P\left(n\right)=\frac{l^{l}}{\Gamma \left(l\right)}\cdot n^{l-1}\cdot \exp\left(-ln\right)$$

where l is the number of looks of the SAR image, exp(·) represents the exponential function, and Γ(·) represents the Gamma function.

Taking a d-dimensional testing sample $y={\left[y_{1},y_{2},\ldots ,y_{d}\right]}^{T}$ as an example, the corresponding speckle can be expressed by $\varepsilon_{i}={\left[n_{i1},n_{i2},\ldots ,n_{id}\right]}^{T}$, where ${}^{T}$ represents the matrix transposition. Using Equation (1), we have $y_{c}=r_{ic}\cdot n_{ic}$, where $y_{c}$ is the cth element of y, $n_{ic}$ is the cth element of $\varepsilon_{i}$ (c=1,2,…,d), and $R_{i}=D_{i}\alpha_{i}={\left[r_{i1},r_{i2},\ldots ,r_{id}\right]}^{T}$. Following Equation (5), $p\left(y_{c}|\alpha_{i}\right)$ can be established as

(6) $$p\left(y_{c}|\alpha_{i}\right)=\frac{l^{l}{\left(y_{c}\right)}^{l-1}}{\Gamma \left(l\right)}\frac{1}{{\left(r_{ic}\right)}^{l}}\exp\left(-l\frac{y_{c}}{r_{ic}}\right)$$

Additionally, $p\left(y|\alpha_{i}\right)$ can be given by

(7) $$p\left(y|\alpha_{i}\right)={\left[\frac{l^{l}}{\Gamma \left(l\right)}\right]}^{d}\prod_{c=1}^{d}\frac{{\left(y_{c}\right)}^{l-1}}{{\left(r_{ic}\right)}^{l}}\exp\left(-\sum_{c=1}^{d}l\frac{y_{c}}{r_{ic}}\right)$$

Here, we have obtained the likelihood function $p\left(y|\alpha_{i}\right)$. In the following, we come to deduce the prior function $p\left(\alpha_{i}\right)$. To guarantee the sparsity of $\alpha_{i}$, $p\left(\alpha_{i}\right)$ is constructed by the Laplace distribution [49].

(8) $$p\left(\alpha_{i}\right)\propto \exp\left(-\eta {\left\|\alpha_{i}\right\|}_{1}\right)$$

where η is a constant, which can be used as a regulator.

From the objective function shown by Equation (4), we can tell that we would like $p\left(\alpha_{i}\right)$ to be large enough to obtain an accurate sparse representation of the SAR image. From Equation (8), we know that a large $p\left(\alpha_{i}\right)$ will lead to a small ${\left\|\alpha_{i}\right\|}_{1}$. Meanwhile, a small ${\left\|\alpha_{i}\right\|}_{1}$ means that most entries of $\alpha_{i}$ are close to zero. Said another way, the sparsity of the representation is guaranteed by the prior function.

Substituting Equations (7) and (8) into Equation (4), the objective function can be expressed as

(9) $$\begin{array}{cc} J_{i} & =\underset{\alpha_{i}}{\arg\max}\;p\left(y|\alpha_{i}\right)p\left(\alpha_{i}\right)\\ & \propto \underset{\alpha_{i}}{\arg\max}\left[\ln p\left(y|\alpha_{i}\right)+\ln p\left(\alpha_{i}\right)\right]\\ & =\underset{\alpha_{i}}{\arg\max}\ln\left\{{\left[\frac{l^{l}}{\Gamma \left(l\right)}\right]}^{d}\prod_{c=1}^{d}\frac{{\left(y_{c}\right)}^{l-1}}{{\left(r_{ic}\right)}^{l}}\exp\left(-\sum_{c=1}^{d}l\frac{y_{c}}{r_{ic}}\right)\right\}-\eta {\left\|\alpha_{i}\right\|}_{1}\\ & \propto \underset{\alpha_{i}}{\arg\max}l\left[\sum_{c=1}^{d}\left(\ln\frac{1}{r_{ic}}-\frac{y_{c}}{r_{ic}}\right)\right]-\eta {\left\|\alpha_{i}\right\|}_{1} \end{array}$$

Inspecting Equation (9), we can see that the objective function consists of two terms. The first one ensures the accurate description of the SAR image and the other one guarantees the sparsity of the description.

As is discussed for the SR-based algorithms, the most important point is to obtain the sparse vector of the SAR image. We can get the sparse vector of the testing sample by optimizing Equation (9). However, it is obvious that the problem of solving Equation (9) is not convex. We can solve it by using the multi-stage convex relaxation method presented in Reference [50].

**Figure 1:**
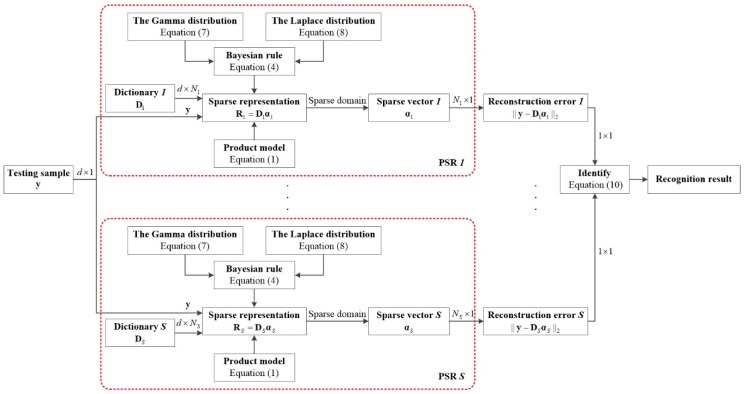
The flow diagram of the proposed algorithm.

Implementing similar processing procedures expressed by Equations (2)–(9) to all the S dictionaries, we can get S different sparse vectors. For all the S vectors, each one will give its description to the testing sample. The one that best describes the testing sample will be the recognition result. That is to say, we will identify the label of the testing sample y by finding the minimum reconstruction error, which can be formulated as

(10) $$L(y)=\underset{i}{\arg\min}\|y-D_{i}\alpha_{i}{\|}_{2}$$

where L(y) represents the label of y. The flow diagram of the proposed algorithm is demonstrated in Figure 1.

## 3. Experimental Results and Analysis

The standard MSTAR database [39,40] is used to test the performance of the proposed algorithm. The SAR images collected with depression angles of 17° and 15° are used as the training datasets and testing datasets, respectively. The datasets consist of 10 different types (the BMP2-tank, BTR70-armored car, T72-tank, BTR60-armored car, 2S1-cannon, BRDM2-truck, D7-bulldozer, T62-tank, ZIL131-truck, and ZSU23/4-cannon) with 14 configurations. All the acronyms of the targets represent different Soviet equipment. The BMP2-tank stands for “Boyevaya Mashina Pekhoty”, both the BTR70-armored car and BTR60-armored car stand for ”Bronetransporter”, the T72-tank and T62-tank are a family of Soviet main battle tanks, the 2S1-cannon stands for “2С1 Carnation”, the BRDM2-truck stands for “Boyevaya Razvedyvatelnaya Dozornaya Mashina”, the D7-bulldozer stands for “Doobi”, the ZIL131-truck stands for “Zavod Imeni Likhachova”, and the ZSU23/4-cannon stands for “Zenitnaya Samokhodnaya Ustanovka”. Detailed information on the training and testing samples is given in Table 1. From Table 1, we can see that the BMP2 tank is comprised of three different configurations, which are BMP2-9563, BMP2-9566, and BMP2-C21, respectively. The T72 tank is also comprised of three different configurations, which are T72-132, T72-812, and T72-S7, respectively. The size of the original SAR image is 128 pixels × 128 pixels, and the aspect angles of the targets lie between 0°–360°.

Firstly, a 60 pixels × 60 pixels sub-image is extracted from the center of each image. Then the intensity of the extracted sub-image is normalized. Dimensionality reduction is realized by adopting the independent and identically distributed Gaussian random matrix [29]. The parameter η in Equation (8) is chosen from the set $\left\{10^{-2},10^{-1},10^{0},10^{1},10^{2}\right\}$ by using the 5-fold cross-validation. We will test the performance of the proposed algorithm by the following experiments. Firstly, we will test its performance by type recognition just as with the existing algorithms. Then, we will further verify the effectiveness of the proposed algorithm on configuration recognition with comparisons to some of the state-of-the-art algorithms.

### 3.1. Type Recognition

#### 3.1.1. 3-Type Recognition

In the beginning, we test the proposed algorithm on 3-type recognition [31,32,38], which include the BMP2 tank, BTR70 armored car, and T72 tank. For the targets with more than one configurations (BMP2 and T72), only the BMP2-9563 and T72-132 are chosen to be the training samples, and all the 3 configurations are selected to be the testing samples. The classic k nearest neighbor (k-NN) [51] and support vector machine (SVM) [52] algorithms are used as competitors. Besides, the SR-based algorithm [29], and some of its modifications (MSR [34], JSR [31] and the label-dependent sparse representation (LSR) [40]) are also used to compare the performance of the proposed algorithm. Recognition results under different algorithms are tabulated in Table 2.

As can be seen from Table 2, the SR-based algorithms perform much better than the traditional k-NN and SVM, which verifies the advantages of sparse representation. As for the other algorithms, MSR utilizes the monogenic signal as the feature to realize recognition. JSR makes use of multi-view samples to improve performance. Finally, LSR achieves recognition by combining the sparse representation and the Gaussian mixture distribution. In LSR, SAR images are described by the additive model, and the residual error term is modeled by the Gaussian mixture distribution. As can be seen, all the SR-based algorithms can obtain satisfying results. As for the proposed PSR, thanks to the precise descriptions and the essential characteristics of the SAR images captured by using the product model, the SAR images are described more accurately than by using the additive model. Moreover, the influences of the speckle can be effectively weakened through the utilization of the Gamma distribution. Satisfying recognition results are obtained by using the proposed algorithm. It can get the best recognition results from all the competitors. The proposed algorithm can achieve an average accurate recognition rate of 96.92% for 3-type recognition, which is 18.97%, 14.14%, 6.08%, 3.44%, 2.63%, and 1.17% better than the competitors. Furthermore, comparing the results of LSR and PSR, we can see that more accurate descriptions of the SAR images lead to better recognition.

#### 3.1.2. 10-Type Recognition

What follows is to realize 10-type recognition, which is much more difficult than 3-type recognition. Similar to the 3-type recognition, different algorithms are utilized as competitors to compare the performance of the proposed algorithm. The BMP2-9563 and T72-132 are chosen to be the training samples for BMP2 and T72, respectively. All the testing samples of BMP2 and T72 demonstrated in Table 1 are used for testing. The corresponding recognition results are given in Table 3.

Similarly, we can see that the SR-based recognition algorithms are much more powerful than k-NN and SVM. All the SR-based algorithms can achieve the recognition rate of more than 90%. The proposed PSR still performs the best in this experiment. It can achieve an average recognition rate of as high as 95.54%, which is 20.58%, 14.87%, 5.37%, 3.31%, 2.56%, and 1.19% higher than k-NN, SVM, SR, MSR, JSR, and LSR, respectively.

### 3.2. Configuration Recognition

#### 3.2.1. 3-Configuration Recognition

**Figure 2:**
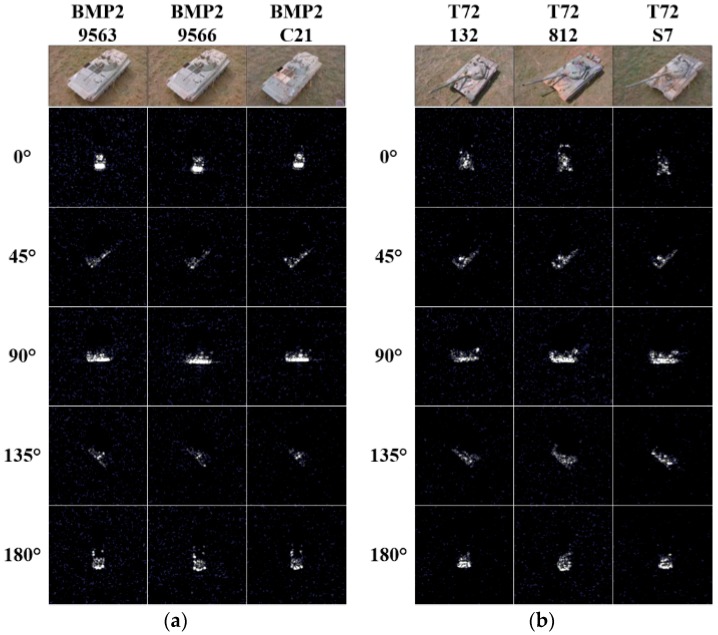
The optical images and SAR images of the targets. (**a**) BMP2 configurations; (**b**) T72 configurations.

From this part, we come to discuss what we focus on most in this paper—configuration recognition. Different from the traditional SAR target recognition algorithms [31,32,38], in which misjudging one configuration of one type into another configuration of the same type is regarded as a right judgment, the proposed algorithm aims to distinguish the tiny differences within the same target type. In other words, different configurations within the same type will be regarded as different targets in this experiment. Configuration recognition is much more challenging than type recognition. All the training and testing samples of BMP2 and T72 demonstrated in Table 1 are utilized for BMP2 configurations and T72 configurations, respectively. Figure 2 demonstrates the optical images and the corresponding SAR images of BMP2 configurations and T72 configurations. In Figure 2, each column represents a certain target with different target aspect angles. Since SAR images are sensitive to target aspect angles owing to the shadowing effects, the interaction of the signature with the environment, the projection of a three-dimensional (3D) scene onto a slant plane, and other reasons related to the aspect dependence of RCSs, they appear quite different with different target aspect angles [4]. Taking any target as an example, e.g., BMP2-9563 in Figure 2a, we can see that the differences of the SAR images are very explicit along the column direction. However, we can see that differences among different configurations with the same target aspect angle are very small (along the row direction). In this challenging experiment, only the well performed SR-based algorithms are utilized to realize recognition.

**Figure 3:**
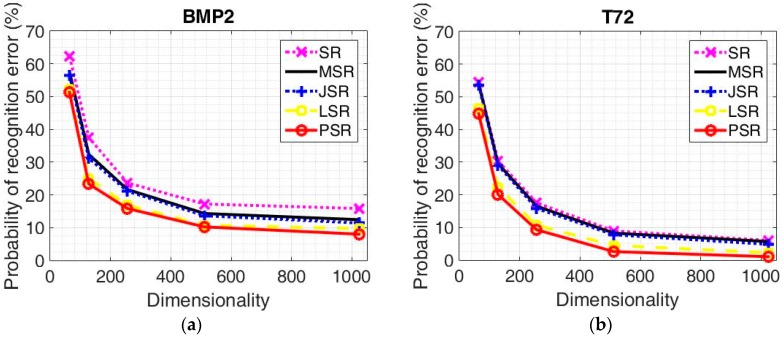
The probability of a recognition error under the different algorithms with different dimensionalities. (**a**) BMP2 configurations; (**b**) T72 configurations.

Recognition results under different algorithms for BMP2 configurations are displayed in Table 4 with feature dimensionalities 64, 128, 256, 512, and 1024, respectively. The corresponding recognition results for T72 configurations are given in Table 5. As can be seen, with the increasing of the feature dimensionality, the performance of all the algorithms gets better accordingly. This is due to the fact that more useful information is kept by a larger feature dimensionality. From the experimental results, we can see that all the algorithms can achieve the best recognition rates with the 1024 feature dimensionality. The proposed PSR can achieve the average accurate recognition rate of 91.99% for BMP2 configurations, which is 7.83%, 4.43%, 3.40%, and 1.70% better than the competitors, respectively. As for the T72 configurations, the recognition rate of the proposed PSR is 97.94%, which is 4.98%, 4.64%, 3.44%, and 1.38% better than the competitors, respectively. The accurate recognition rates of each target under the 1024 feature dimensionality are displayed in Table 6 and Table 7 for BMP2 configurations and T72 configurations, respectively. The probability of recognition error versus feature dimensionality for BMP2 configurations and T72 configurations under different algorithms are shown in Figure 3a,b, respectively. The probability of a recognition error of the proposed algorithm is the lowest for both BMP2 configurations and T72 configurations in all the dimensionalities. As can be seen, the performance of the T72 configurations is much better than that of BMP2 configurations for all the given dimensionalities. This is due to the fact that the BMP2 configurations are much more similar to each other with respect to the T72 configurations [53]. Still, we focus on the BMP2 configuration recognition which is more difficult. We can tell that from Figure 3a, only LSR and PSR can achieve an accurate recognition rate of nearly 90% with feature dimensionality 512. Although MSR and JSR can achieve much better performance than SR, they lose competitiveness with respect to the statistical model based recognition algorithms. This further verifies the fact that taking the essential characteristics of SAR images into consideration is helpful for recognition. The comparison between LSR and PSR demonstrates that better descriptions of the SAR image and the noise component lead to better recognition results.

**Figure 4:**
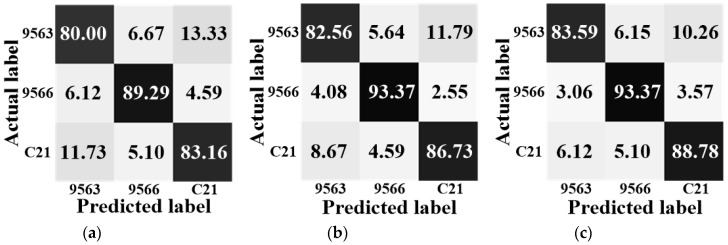

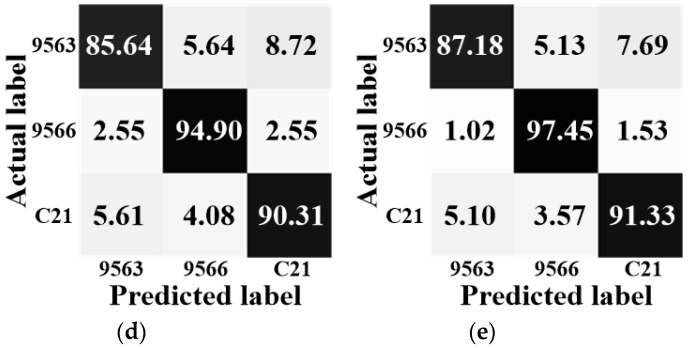
The confusion matrices for the BMP2 configurations. (**a**) SR; (**b**) MSR; (**c**) JSR; (**d**) LSR; (**e**) PSR.

**Figure 5:**
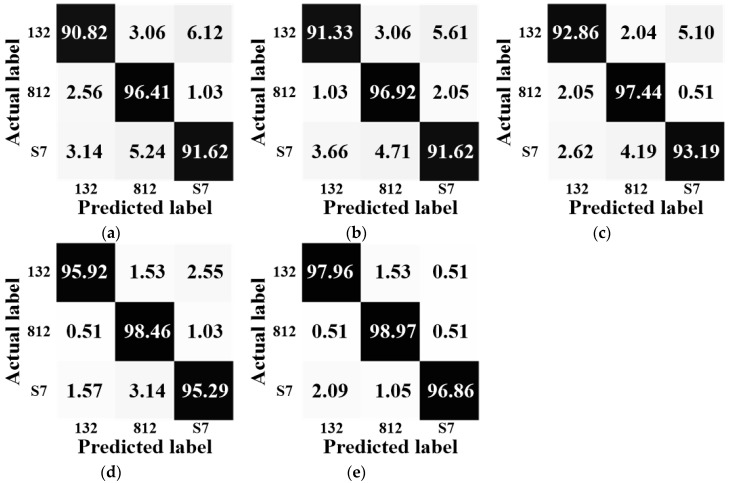
The confusion matrices for the T72 configurations. (**a**) SR; (**b**) MSR; (**c**) JSR; (**d**) LSR; (**e**) PSR.

To see the details of how the target is recognized, confusion matrices under different algorithms with the 1024 feature dimensionality for BMP2 configurations and T72 configurations are shown in Figure 4 and Figure 5, respectively. Here, we give a brief discussion about the information shown by these matrices. In Figure 4 and Figure 5, the horizontal axis represents the predicted label and the vertical axis represents the actual label. Taking BMP2 as an example, in Figure 4a, the big matrix consists of nine small boxes, the left-top one shows that the rate of recognizing the BMP2-9563 sample into the BMP2-9563 category (the accurate recognition rate) is 80.00%, the middle-top one shows that the rate of recognizing the BMP2-9563 sample into the BMP2-9566 category is 6.67%, and the right-top one shows that the rate of recognizing the BMP2-9563 sample into the BMP2-C21 category is 13.33%. We can see that the summation of these rates is 100%. In other words, the values lay along the diagonal direction display the accurate recognition rates under a certain algorithm. Among all the matrices obtained by using different algorithms, the proposed one, i.e., Figure 4e, can achieve the best recognition results.

#### 3.2.2. Configuration Recognition with Random Corruption

**Figure 6:**
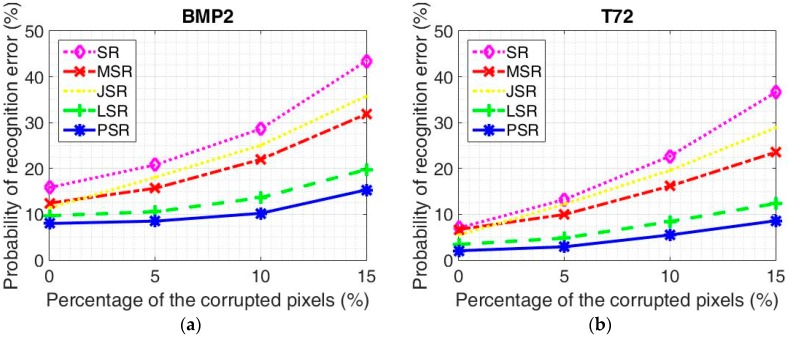
The probability of a recognition error under the different algorithms with different percentages of corruption. (**a**) BMP2 configurations; (**b**) T72 configurations.

In this part, we come to test the robustness of the proposed algorithm under noisy conditions. The SAR images are corrupted in the following way with different corruption percentages. In the beginning, a percentage of pixels of the image are randomly picked out, and then the corresponding positions are filled with independent and identically distributed samples which obey a uniform distribution [29]. The experimental datasets are exactly the same as what is used in Section 3.2.1. The probability of a recognition error under different algorithms for BMP2 configurations is given in Figure 6a, whereas the corresponding results for the T72 configurations are displayed in Figure 6b. From the results, we can see that the performance of all the algorithms will deteriorate with the increase of the corruption percentage from 0% to 15%.

Firstly, we come to see the robustness of the algorithms in the following way for both of the datasets. We calculate the increment of the recognition error with each 5% increase of the corruption percentage. For BMP2 configurations, the recognition error increment of SR is 4.94%, 7.84%, and 14.82% for each 5% increase. The recognition error increment of MSR is 3.23%, 6.30%, and 9.88% for each 5% increase. The recognition error increment of JSR is 6.65%, 6.98%, and 10.73% for each 5% increase. The recognition error increment of LSR is 0.85%, 3.07%, and 6.13% for each 5% increase. The recognition error increment of the proposed PSR is 0.51%, 1.70%, and 5.11% for each 5% increase.

For T72 configurations, the recognition error increment of SR is 6.19%, 9.45%, and 13.92% for each 5% increase. The recognition error increment of MSR is 3.26%, 6.19%, and 7.39% for each 5% increase. The recognition error increment of JSR is 6.70%, 7.39%, and 9.28% for each 5% increase. The recognition error increment of LSR is 1.37%, 3.61%, and 3.95% for each 5% increase. The recognition error increment of the proposed PSR is 0.86%, 2.58%, and 3.09% for each 5% increase.

Thanks to the accurate descriptions of SAR images, LSR and PSR can achieve much better recognition results than the competitors. From the results, we can tell that due to the better descriptions of the SAR images and the use of the Gamma distribution to model the speckle component, the recognition error increments of the proposed PSR is much lower for both of the two datasets, which further proves the robustness of the proposed PSR under noisy conditions.

#### 3.2.3. Recognition of Eight Different T72 Configurations

To further validate the effectiveness and advantage of the proposed algorithm, we have conducted another challenging experiment, in which eight different T72 configurations (T72-A04, T72-A05, T72-A07, T72-A10, T72-A32, T72-A62, T72-A63, and T72-A64) are recognized. This case is very difficult to realize since all the T72 targets are quite similar to each other except for some tiny differences, such as with/without spotlights, splash guard, etc. The datasets description of the eight different T72 configurations is displayed in Table 8. The same as other experiments, the samples with the depression angle of 17° are used as the training samples, whereas the ones with the depression angle of 15° are used as the testing ones. Corresponding recognition results under various algorithms are given in Table 9. As can be seen, in this severe situation, the performance of all the algorithms will drop dramatically. The algorithms deduced from the statistical view (LSR and PSR) can obtain better results than other SR-based algorithms. From the results, we can tell that taking the characteristics of SAR images into account will contribute to better recognition results. As for the proposed algorithm, it still outperforms all the competitors. Thanks to the better description of the SAR image, essential characteristics of the samples can be obtained. Fusing the advantage into SR technique, the discriminative power has been further enhanced. We can tell that the proposed algorithm can achieve an average recognition rate of 86.43%, which is 5.71%, 3.84%, 2.56%, and 1.28% better than SR, MSR, JSR, and LSR, respectively. In LSR, the SAR images are modeled by the additive model, whereas they are described by the product model by the proposed PSR. The experimental results further demonstrate the fact that SAR images can be better described by the product model than the additive model.

## 4. Conclusions

In this paper, a PSR algorithm is proposed for SAR target configuration recognition. The SAR images are described by the product model and the essential characteristics of SAR images can be captured. Besides, the sparse representation and the product model are combined together, leading to a more accurate sparse description of the SAR image. What is more, the Gamma distribution is employed to model the speckle noise of SAR images and the sparse vector is obtained in the statistical view, which can enhance the robustness of the proposed algorithm under noisy conditions.

The effectiveness of the proposed PSR algorithm is validated on the standard MSTAR database. The accuracies are compared with some advanced recognition algorithms. Satisfying recognition results are obtained. From the experimental results, we can draw the following conclusions. (1) Exploiting the essential characteristics of SAR images and modeling them accurately will lead to exciting recognition rates; (2) The proposed algorithm can achieve satisfying recognition rates in both SAR target type recognition and configuration recognition; (3) The proposed algorithm is robust to noise due to the utilization of the statistical model.

In addition, more advanced statistical models are still in research, how to fuse the advantages into the proposed algorithm is well worth working on.

Moreover, in this paper, we view all the targets in SAR images from the MSTAR database as targets without the consideration of false alarms. In practice, when we aim to recognize the targets in SAR images with much more complicated backgrounds, more factors such as the correct rejection probability should be taken into consideration, since they will highly affect the final recognition results.

## Figures and Tables

**Table 1:** The datasets descriptions.

Training Set (17°)			Testing Set (15°)		
Target		Number of Images	Target		Number of Images
BMP2	9563	233	BMP2	9563	195
	9566	232		9566	196
	C21	233		C21	196
T72	132	232	T72	132	196
	812	231		812	195
	S7	228		S7	191
BTR70		233	BTR70		196
BTR60		256	BTR60		195
2S1		299	2S1		274
BRDM2		298	BRDM2		274
D7		299	D7		274
T62		299	T62		273
ZIL131		299	ZIL131		274
ZSU23/4		299	ZSU23/4		274

**Table 2:** The recognition results for the 3-type recognition.

Algorithm	BMP2	BTR70	T72	Average
k-NN (%)	72.74	83.67	81.27	77.95
SVM (%)	77.17	90.31	85.91	82.78
SR (%)	85.69	94.39	94.85	90.84
MSR (%)	90.46	96.94	95.36	93.48
JSR (%)	90.97	98.47	96.22	94.29
LSR (%)	92.16	100	97.94	95.75
PSR (%)	93.87	100	98.97	96.92

**Table 3:** The recognition results for 10-type recognition.

Algorithm	BMP2	BTR70	T72	BTR60	2S1	BRDM2	D7	T62	ZIL131	ZSU23/4	Average
k-NN (%)	61.50	82.14	70.79	79.49	64.96	85.04	90.15	73.26	83.94	81.75	74.96
SVM (%)	67.63	88.27	77.32	84.62	69.71	91.97	94.89	76.56	88.32	89.42	80.67
SR (%)	80.92	96.94	89.69	94.87	86.50	92.34	97.45	91.58	92.34	93.43	90.17
MSR (%)	81.94	97.45	89.86	96.41	94.89	95.26	97.81	94.14	95.62	95.99	92.23
JSR (%)	82.62	97.45	90.03	96.92	96.72	95.62	98.54	95.97	96.35	97.08	92.98
LSR (%)	83.82	98.98	90.72	97.95	99.64	98.54	99.27	97.44	98.18	97.45	94.35
PSR (%)	85.86	100	92.61	98.97	99.64	98.91	99.64	98.53	98.91	98.91	95.54

**Table 4:** The recognition results for configurations of BMP2.

Feature Dimensionality	64	128	256	512	1024
SR (%)	37.65	62.52	76.32	82.79	84.16
MSR (%)	42.42	67.63	78.36	85.69	87.56
JSR (%)	43.44	68.82	78.88	86.37	88.59
LSR (%)	47.70	74.96	83.13	89.27	90.29
PSR (%)	48.72	76.66	84.16	89.78	91.99

**Table 5:** The recognition results for configurations of T72.

Feature Dimensionality	64	128	256	512	1024
SR (%)	44.85	68.56	81.44	89.69	92.96
MSR (%)	45.19	69.59	82.47	90.72	93.30
JSR (%)	45.88	70.45	82.99	91.24	94.50
LSR (%)	52.92	76.63	88.49	94.50	96.56
PSR (%)	54.30	79.04	89.69	96.39	97.94

**Table 6:** The recognition results of each configuration for BMP2 under the 1024 feature dimensionality.

Algorithm	BMP2	BMP2	BMP2	Average
	9563	9566	C21	
MSR (%)	82.56	93.37	86.73	87.56
JSR (%)	83.59	93.37	88.78	88.59
LSR (%)	85.64	94.90	90.31	90.29
PSR (%)	87.18	97.45	91.33	91.99

**Table 7:** The recognition results of each configuration for T72 under the 1024 feature dimensionality.

Algorithm	T72	T72	T72	Average
	132	812	S7	
MSR (%)	91.33	96.92	91.62	93.30
JSR (%)	92.86	97.44	93.19	94.50
LSR (%)	95.92	98.46	95.29	96.56
PSR (%)	97.96	98.97	96.86	97.94

**Table 8:** The dataset descriptions of the eight T72 configurations.

Configuration	T72	T72	T72	T72	T72	T72	T72	T72
	A04	A05	A07	A10	A32	A62	A63	A64
Testing set (17°)	299	299	299	296	298	299	299	299
Testing set (15°)	274	274	274	271	274	274	274	274

**Table 9:** The recognition results of the eight T72 configurations under different algorithms.

Algorithm	T72	T72	T72	T72	T72	T72	T72	T72	Average
	A04	A05	A07	A10	A32	A62	A63	A64	
SR (%)	72.26	79.20	69.71	90.41	87.59	87.23	79.93	79.56	80.72
MSR (%)	73.36	79.93	75.18	91.88	88.69	89.05	81.02	81.75	82.59
JSR (%)	74.45	80.29	77.37	92.62	89.42	90.88	82.48	83.58	83.87
LSR (%)	76.28	81.75	79.56	94.10	90.51	91.61	83.58	83.94	85.15
PSR (%)	78.83	83.21	82.12	94.46	90.88	92.34	84.31	85.40	86.43
